# Numerical Study on Flow and Heat Transfer Characteristics of Trapezoidal Printed Circuit Heat Exchanger

**DOI:** 10.3390/mi12121589

**Published:** 2021-12-20

**Authors:** Yuxuan Ji, Kaixiang Xing, Kefa Cen, Mingjiang Ni, Haoran Xu, Gang Xiao

**Affiliations:** State Key Laboratory of Clean Energy Utilization, Zhejiang University, 38 Zheda Road, Hangzhou 310027, China; ji_yuxuan@zju.edu.cn (Y.J.); 3120102460@zju.edu.cn (K.X.); kfcen@zju.edu.cn (K.C.); mjn@zju.edu.cn (M.N.); haoranxu@zju.edu.cn (H.X.)

**Keywords:** printed circuit heat exchanger (PCHE), trapezoidal channel, numerical simulation, thermal and hydraulic performance

## Abstract

Printed circuit heat exchanger (PCHE) is a promising regenerative device in the sCO_2_ power cycle, with the advantages of a large specific surface area and compact structure. Its tiny and complex flow channel structure brings enhanced heat transfer performance, while increasing pressure drop losses. It is, thus, important to balance heat transfer and flow resistance performances with the consideration of sCO_2_ as the working agent. Herein, three-dimensional models are built with a full consideration of fluid flow and heat transfer fields. A trapezoidal channel is developed and its thermal–hydraulic performances are compared with the straight, the S-shape, and the zigzag structures. Nusselt numbers and the Fanning friction factors are analyzed with respect to the changes in Reynolds numbers and structure geometric parameters. A sandwiched structure that couples two hot channels with one cold channel is further designed to match the heat transfer capacity and the velocity of sCO_2_ flows between different sides. Through this novel design, we can reduce the pressure drop by 75% and increase the regenerative efficiency by 5%. This work can serve as a solid reference for the design and applications of PCHEs.

## 1. Introduction

Printed circuit heat exchanger (PCHE) is an advanced heat transfer device with millimeter-scale internal flow grooves. The micro-channels are chemically etched on stainless steel plates, and the etched plates are diffusion-bonded to form a connection between metal atoms. The strength of diffusion welding can reach almost the same with the plate material. PCHE possesses advantages of high efficiency, compactness, and robustness at high temperature and pressure conditions. It is a promising choice for various engineering applications, such as the regenerator of the supercritical carbon dioxide (sCO_2_) power cycle. Fluid flow and heat transfer characteristics in PCHE are significantly different from those in traditional shell-and-tube heat exchangers, and its thermal–hydraulic performance has a great impact on the efficiency and output power of the sCO_2_ power cycle due to the huge heat recovery.

Some existing experimental research has tested the performance of PCHE using He, water, or sCO_2_ as the working fluid. Ishizuka et al. [1] studied the heat transfer and pressure drop characteristics of PCHE on the sCO_2_ cycle experimental loop and proposed empirical formulas for pressure loss, and local and overall heat transfer coefficients, with the Reynolds number range of 2400–6000 and 5000–13,000 at the hot and cold side, respectively. Nikitin et al. [2] studied the heat transfer and pressure drop characteristics of a zigzag PCHE. As a result, the experimentally measured overall heat transfer coefficient of PCHE was in the range of 300–650 W/m^2^. Ngo et al. [3,4] studied the thermo-hydraulic characteristics of zigzag and S-shape PCHE using carbon dioxide as the working fluid, and proposed that the Nusselt number of zigzag PCHE was 24–34% higher than that of the S-shape, with a four to five times higher pressure drop under the same Reynolds number. Kim et al. [5,6,7] carried out comprehensive numerical and experimental research on zigzag PCHE using He, water, carbon dioxide, and their mixed working fluids. The correlations of the Fanning friction factor and Nusselt number with respect to the channel angle, pitch length, and hydraulic diameter in the zigzag PCHE were obtained. Mylavarapu et al. [8] designed and manufactured two straight-channel PCHEs on the high-temperature helium test platform. The experimental correlations of the Nusselt number and the Fanning friction factor were obtained, and it was also found that the laminar to turbulent transition region appeared earlier in the PCHE than in the circular tube, corresponding to a Reynolds number of 1700. Baik et al. [9] built a cycle test platform and performed a heat transfer performance test using sCO_2_ and water. The Fanning friction factor and heat transfer correlations were proposed, respectively. Chu et al. [10] also designed a sCO_2_ and water heat transfer platform and tested the performance of a straight channel PCHE. Experiments found that the heat transfer performance of sCO_2_ was 1.2–1.5 times better than that of water. Zhang et at. [11] tested a 100-kW novel airfoil fin PCHE as a cooler, and also conducted the numerical analysis. The airfoil fin showed a comparative heat transfer rate with only 1/6 pressure drop of the zigzag structure.

Table 1 summarizes these existing experiments, from which it can be found that most experiments have the defect of low parameter conditions in pressure and temperature, and only one-type facilities can be tested due to the complicated manufacturing process of PCHE. Although experimental research can accurately reflect the performance of PCHE, the difficulty of manufacturing and high cost constrains the extensive development. Numerical simulation can be used to design and optimize PCHE channels quickly and effectively with lower costs and relatively reliable accuracy compared with experiments. The parameters of temperature and pressure can reach the industrial level and the local flow and heat transfer characteristics inside the PCHE micro-flow channels can be obtained.

Some numerical simulation studies are listed in Table 2, and the comparisons with the existing experiments proved the credibility of the simulation results. Tsuzuki et al. [12] established a three-dimensional S-shaped PCHE model and concluded that the S-fin flow channel can reach the same thermal performance as the zigzag flow channel, but its pressure loss reduction can be reduced to one-fifth. Furthermore, they also studied the influence of the fin shape structure on the thermal and hydraulic performance through CFD simulation [13], and proposed the correlation of the Nusselt number in the PCHE of the S-shaped flow channel structure [14]. Kim et al. [15] established a three-dimensional sCO_2_ zigzag PCHE model and performed related numerical simulations. It was found that the outlet temperature and pressure obtained from the simulation agreed well with the experimental results of Ishizuka et al. In addition, they proposed a structure of airfoil ribs, which can reduce the pressure drop loss to one-twelfth of the zigzag channel, while ensuring the heat exchange performance. Bartel et al. [16] conducted a series of comparative studies on the zigzag flow channel PCHE and found that the best heat transfer performance was obtained at a pitch angle of 15°, with the increase in pressure drop loss acceptable relative to the straight-channel PCHE. Khan et al. [17] performed a three-dimensional steady-state heat transfer simulation of zigzag PCHE at four different inclination angles of 0°, 5°, 10°, and 15° and four different Reynolds numbers of 350, 700, 1400, and 2100, which found that there were flow enhancement and secondary flow areas inside the zigzag channel. Kim et al. [18] used the ANSYS CFX to explore and verify the existing zigzag PCHE correlations, and expanded the applicable Reynolds number range to 2000–58,000 based on the Ishizuka’s experimental correlations. Baik et al. [19] numerically studied the S-shape PCHE and analyzed the influence of the channel amplitude and period on the heat transfer performance. Chen et al. [20] compared the performance of four types of NACA 00XX airfoil structures with zigzag and found that the airfoil structure can significantly reduce the flow pressure drop loss while maintaining heat transfer performance. Aneesh et al. [21] used helium working fluid to study the heat transfer performance of zigzag, sine-shaped, and trapezoidal flow channel structures and found that trapezoidal PCHE has the highest heat transfer performance and maximum pressure. Ren et al. [22] developed a new local heat transfer correlation based on a generalized mean temperature difference (GMTD) method in a horizontal semicircular straight channel of PCHE, which predicts 93% of the data, with errors of less than ±15%. Lv et al. [23] proposed three new hybrid flow channel structures, which combined the S-shape in the high-, medium-, and low-temperature sections of the straight channel, respectively. The results showed that the type C (with the wavy section used in the low-temperature region) was the best, with a maximum pressure drop reduction of 23%, and the heat transfer coefficient increased by 2.6 times higher compared to type A (with the wavy section used in the high-temperature region).

Most of the above numerical simulation research conditions deviate from the actual sCO_2_ cycle and lack comparison of different channel structures. Among these structures, the trapezoidal structure presented better heat transfer performance; however, it suffers from a larger pressure drop. To overcome this problem, numerical models of the trapezoidal flow channel are developed, and its thermal and hydraulic performance with sCO_2_ as the medium is studied. The dimensionless Nusselt numbers and Fanning friction factors are analyzed with respect to the changes in Reynolds numbers and structure geometric parameters. An optimized structure is further proposed to match the heat transfer capacity and velocity of hot and cold flows of sCO_2_ at different temperatures and pressures.

## 2. Model and Methods

### 2.1. Model and System

**Figure 1:**
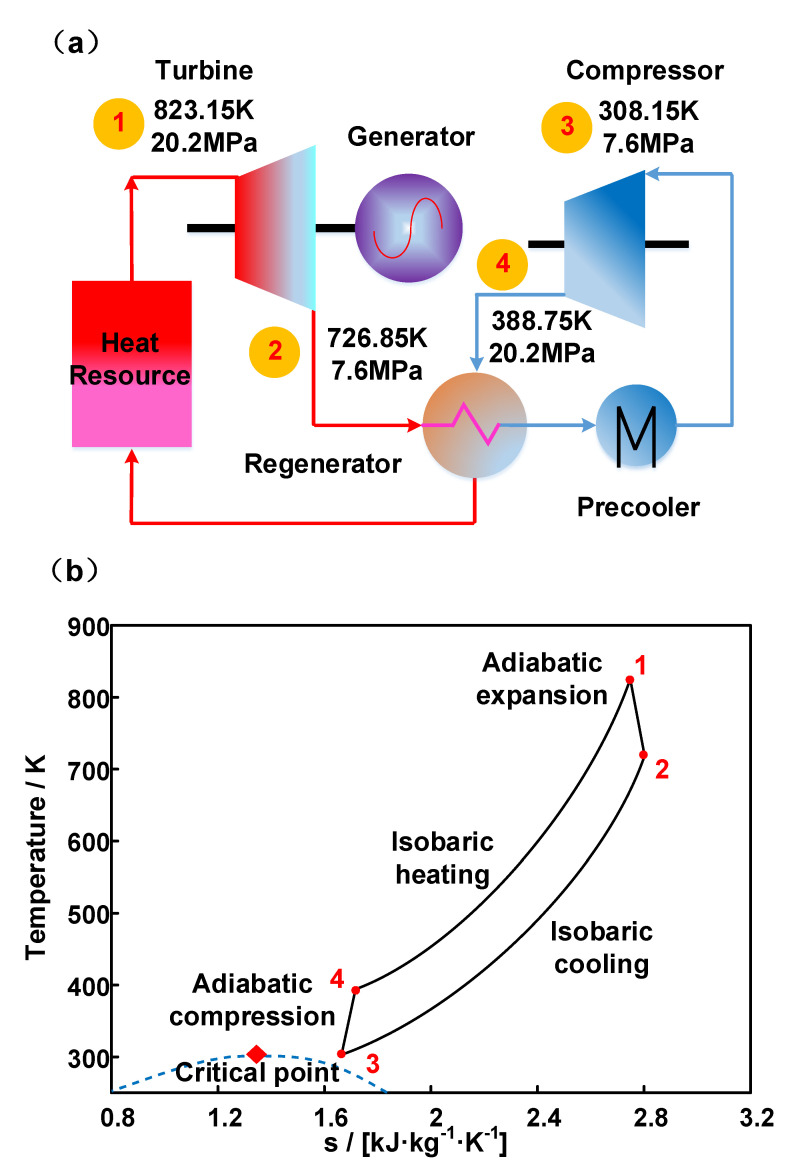
Diagrams of (**a**) a sCO_2_ simple Brayton cycle process and (**b**) the T–s diagram.

The schematic of a simple sCO_2_ Brayton cycle and related temperature–entropy (T-s) diagram are presented in Figure 1 [24]. The high-temperature and high-pressure sCO_2_ from heat resource (point 1) enters the turbine for power generation. After the temperature and pressure reduced (point 2), the medium enters the regenerator to transfer the residual heat to the cold-side working fluid. In addition, after the cooling device, the sCO_2_ with its temperature close to the critical point (point 3) enters the compressor to increase pressure. Finally, the high-pressure medium (point 4) returns to point 1 state by heat recovery and resource heating. Between the cooling and heating processes, a huge amount of heat exchange through the regenerator is needed, making the performance of PCHE vital to the system efficiency.

**Figure 2:**
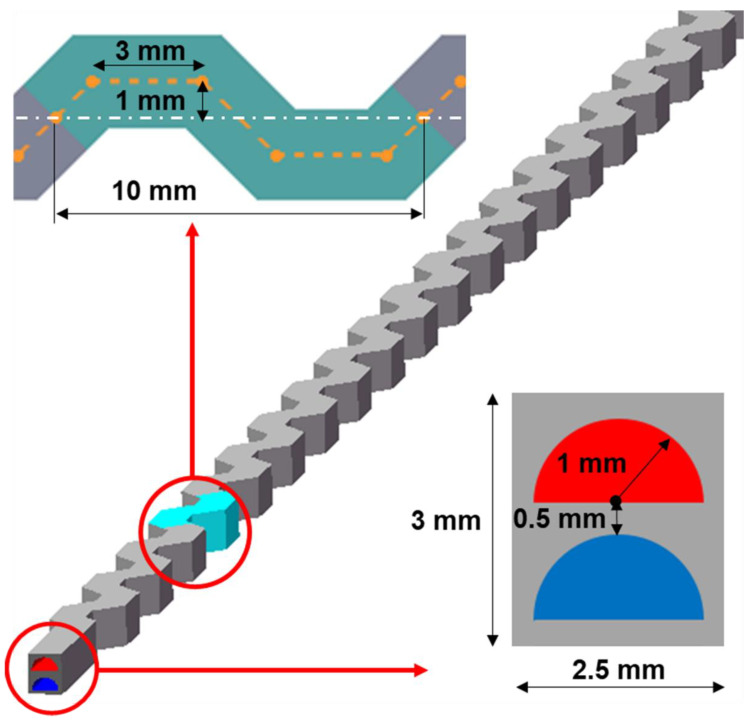
Unit model of channel structure for the numerical simulation.

In this work, a counterflow double-channel PCHE model was established with two channels corresponding to the hot and the cold side. As shown in Figure 2, the channels have semicircular cross-sections with 2 mm diameters and a 0.5 mm space in between. The simulations are completed under the ANSYS software system, in which ICEM is used for meshing and Fluent is used for calculations. The unstructured tetrahedral mesh is divided and 10 bidirectional prism layers are generated in the wall boundary layer. The SST k-omega turbulence model is chosen due to the Reynolds number range and flow bending. The dimensionless y+ is set close to 1 to better reflect the influence of the boundary layer near the wall. Periodic boundary conditions were applied on the top, the bottom, the left, and the right sides, and adiabatic boundary conditions were applied on the front and the back surfaces. The inlet temperature and pressure boundary conditions on the hot and cold channels were 726.85 K, 7.6 MPa and 388.75 K, 20.2 MPa, respectively. Both are far away from the CO_2_ critical point (304.25 K, 7.38 MPa). The inlet mass flow rate range on both sides is from 4.82 × 10^−4^ to 14.45 × 10^−3^ kg/s.

### 2.2. Grid and Independence Verification

To save computing resources while keeping a sufficient accuracy, the grid independence verification was studied using the grid convergence index (GCI). Three grids are selected with a constant refinement ratio and the hot outlet temperature is chosen as a parameter indicative of grid convergence.

For tetrahedral mesh, the effective grid refinement ratio is defined as Equation (1):

(1) $$r={\left(\frac{N_{1}}{N_{2}}\right)}^{\left(\frac{1}{D}\right)}\;$$

Here, N is the total number of grid points and D is the dimension of the flow domain.

The order of convergence is calculated using Equation (2):

(2) $$p=\ln\left(\frac{T_{3}-T_{2}}{T_{2}-T_{1}}\right)/\ln\left(r\right)\;$$

Here, $T_{i}$ are the hot outlet temperature solutions at different meshes, and $T_{1}$ corresponds to the fine grid.

The grid convergence index (GCI) is calculated using Equation (3):

(3) $$GCI_{fine}=\frac{F_{s}\left|\frac{T_{2}-T_{1}}{T_{1}}\right|}{r^{p}-1}\;$$

Here, $F_{s}$ is a safety factor, and the recommended value is 1.25 for three or more grid comparisons.

The checking asymptotic range of convergence is defined by Equation (4):

(4) $$\frac{GCI_{2,\;3}}{r^{p}\times GCI_{1,\;2}}\sim 1\;$$

As shown in Table 3, the $GCI{\;}_{fine}=2.42\%$ and the checking asymptotic range of convergence is 1.0107, which is approximately 1. Therefore, the number 3,780,000 was adopted for meshing in this work.

The following assumptions are adopted:

1 The continuous medium flows uniformly in every channel of the PCHE.
2 The total mass flow of sCO_2_ is distributed equally in each hot/cold channel because the flow resistance is the same.
3 The inlet temperature and pressure of all hot/cold channels are the same and identical to the hot/cold pipe of the PCHE.
4 The effect of pressure changes on the CO_2_ properties is neglectable in the flowing process, as the pressure loss is much smaller than the working pressure.

### 2.3. Properties of sCO_2_

Based on the assumptions above, the properties of sCO_2_ are calculated at pressures of the hot side (7.6 MPa) and the cold side (20.2 MPa), respectively. The thermal and hydraulic properties, including the density, the specific heat, the thermal conductivity, and the dynamic viscosity, can be described as polynomial functions of temperature using MATLAB software between 350 K and 750 K. The purpose is to ensure the accuracy of physical property parameters while reducing the computational resources generated by quoting the FLUENT’s built-in database. All the data are obtained from NIST Reference Database, and the fitting correlations are listed in Table 4.

### 2.4. Calculation Method

To evaluate the thermal and hydraulic performance of the trapezoidal channel, the average heat transfer coefficient ($\bar{h}$) and the Fanning friction factor (f) are calculated at different temperatures and pressures.

$\bar{h}$ is a function of heat flux and temperature difference as expressed in Equation (5):

(5) $$\bar{h}=\frac{q}{T_{w}-T_{b}}$$

Here, q is the heat flux, $T_{w}$ is the area average wall temperature of fluid channel, and $T_{b}$ is the bulk mean temperature of sCO_2_.

The average dimensionless heat transfer coefficient ($\bar{Nu}$) can be further calculated by Equation (6):

(6) $$\bar{Nu}=\frac{\bar{h}d_{h}}{k}$$

Here, $d_{h}$ is the hydraulic diameter of the semicircle cross-section, and k is the thermal conductivity.

f can be calculated as shown in Equation (7):

(7) $$f=\frac{2\Delta p_{f}d_{h}}{\rho lu^{2}}$$

Here, $\Delta p_{f}$ is the pressure loss caused by friction, ρ is the bulk density of sCO_2_, l is the length of trapezoidal flow channel, and u is the bulk velocity. As the density of sCO_2_ changes in both sides with the heat exchange and there exist differences in the velocities and the dynamic pressures, $\Delta p_{f}$ should be calculated as shown in Equation (8):

(8) $$\Delta p_{f}=\Delta P-\left(\left|\frac{1}{2}\rho_{out}u_{out}^{2}-\frac{1}{2}\rho_{in}u_{in}^{2}\right|\right)$$

Here, ΔP is the numerical pressure drop result, and $u_{in}$ and $u_{out}$ are the velocities at the channel inlet and outlet, respectively.

To compare the comprehensive thermal and hydraulic performances of different structures, the performance evaluation criteria (PEC) is defined as [25] shown in Equation (9):

(9) $$\mathrm{PEC}=\frac{\frac{{\bar{Nu}}_{i}}{{\bar{Nu}}_{j}}}{{\left(\frac{f_{i}}{f_{j}}\right)}^{\frac{1}{3}}}$$

Here, the subscripts i and j represent two different structures.

Heat recovery efficiency η is introduced to measure the performance of energy recovery as shown in Equation (10):

(10) $$\eta =\frac{Q_{c}}{Q_{h}}=\frac{m_{c}\left(H_{c,out}-H_{c,in}\right)}{m_{h}\left(H_{h,in}-H_{h,out}\right)}$$

Here, $Q_{c}$ and $Q_{h}$ are the heat fluxes of the cold and the hot channels, respectively; $m_{c}$ and $m_{h}$ are the mass flow rates of the cold and the hot channels, respectively; H represents the enthalpy, and subscripts in and out are the inlet and the outlet of channels, respectively.

## 3. Results and Discussion

### 3.1. Thermal and Hydraulic Performance

The thermal performance of PCHE is evaluated by the mean convective heat transfer coefficients of both sides and the regenerative efficiency, where the dimensionless results are used to analyze the tendency with respect to the changes in geometric parameters, including the flow length, the trapezoidal bottom angle, and the straight length of trapezoidal upper.

**Figure 3:**
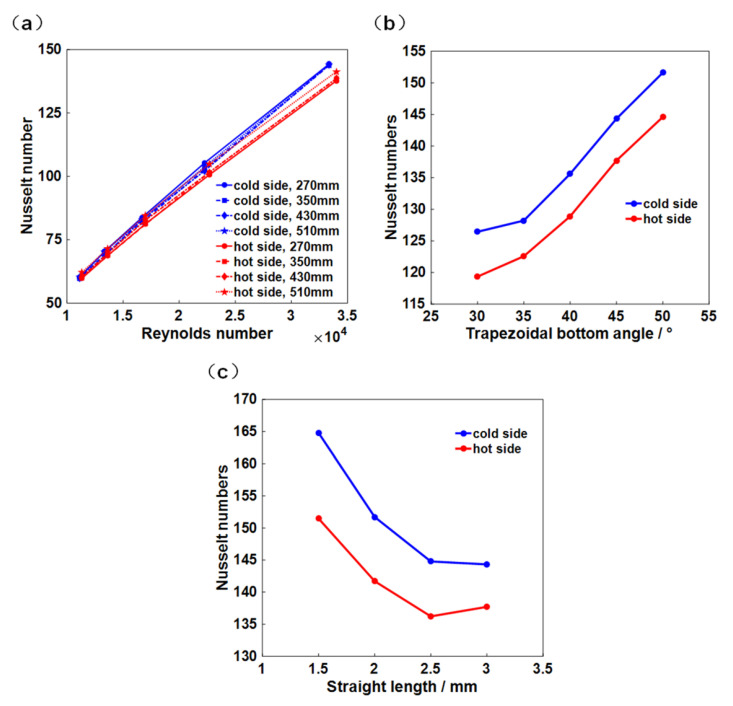
Effects of (**a**) the Reynolds numbers, (**b**) the trapezoidal bottom angle, and (**c**) the straight length on the Nusselt numbers.

The relationship between Nusselt numbers and Reynolds numbers in different length channels are shown in Figure 3a. Both hot and cold channels present linear increase tendencies of Nusselt numbers with the increase in Reynolds numbers. It is worth noting that the Nusselt numbers of different flow lengths are almost the same at the same Reynolds number, indicating the independence of heat transfer performance from the flow length. The Nusselt numbers on both channels increase with the increase in trapezoidal bottom angle, as shown in Figure 3b, indicating a significant improvement in heat transfer performance. Compared with the hot side, larger Nusselt numbers are observed in the cold side, indicating its larger heat transfer capacity. The impact of the straight length of trapezoidal upper on the Nusselt number is shown in Figure 3c. With the increase in channel length from 1.5 mm to 2.5 mm, a significant decline in the Nusselt numbers is observed at the hot side and the cold side, respectively. When it further increases to 3 mm, the Nusselt number at the cold side remains almost unchanged, while it shows a slight increase at the hot side. It can be inferred that the increase in trapezoidal upper length has little effect on the heat transfer performance at more than 3 mm.

**Figure 4:**
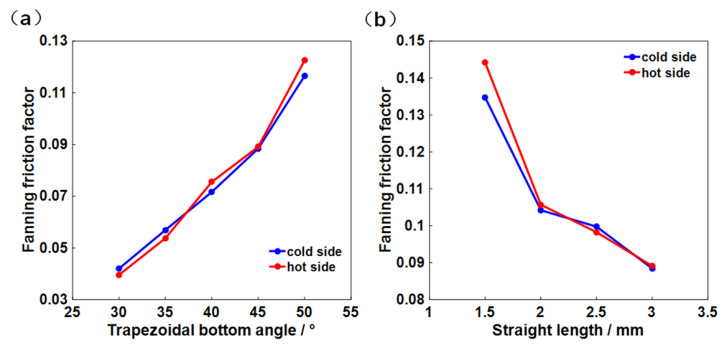
Effects of (**a**) the trapezoidal bottom angle and (**b**) the straight length on the Fanning factors.

The hydraulic performance of the trapezoidal channel in different geometric parameters are compared using the dimensionless Fanning friction factor (f), as shown in Figure 4a. With the increase in the trapezoidal bottom angle, significant increases in f in both the hot side and the cold side are observed, where the values at the 50° bottom angle are about three times larger than those at 30° cases. This can be explained by the increase in the flow vortex at the bend of the channel, resulting in a larger low-momentum flow region and an increased flow resistance.

Fanning friction factor is also affected by the straight length of the trapezoidal upper also, as shown in Figure 4b. Since the flow is more stable in the straight channel and the vortex caused by bending is weakened, trapezoidal channel structure with a longer straight length has the lower f value. In addition, the difference between the hot and cold sides gradually decreases with the increase in straight length.

### 3.2. Comparison between the Trapezoidal and Previous Channel Structures

**Figure 5:**
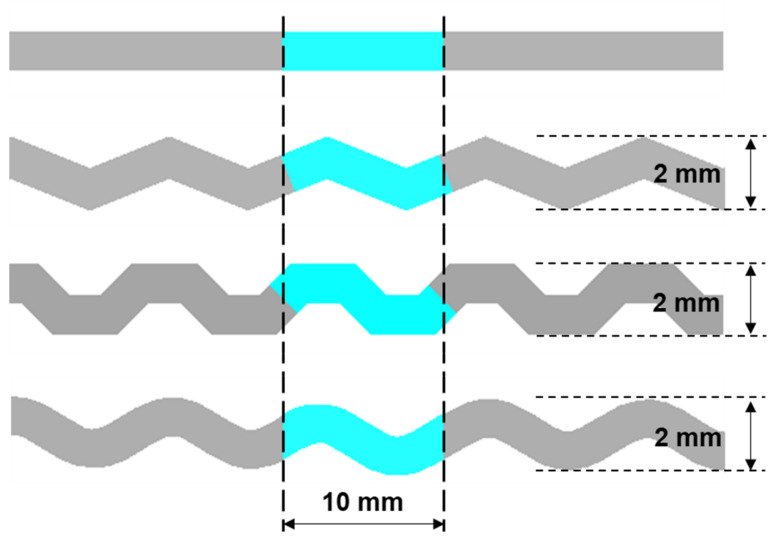
Channel models of the straight, the zigzag, the S-shape, and the trapezoidal structures.

Heat transfer capacity and pressure loss characteristics of the trapezoidal channel are further compared with the previously developed channel structures (i.e., the straight, the zigzag, and the S-shape) by establishing models and numerically simulating, as shown in Figure 5. These models have a 4 mm × 3 mm cross-section and a length of 250 mm in the flow direction with the same period (10 mm) and amplitude (1 mm). The same temperature and pressure boundary conditions are applied as in Section 2.1, and all inlet mass flow rates are set to 14.45 × 10^−3^ kg/s. The four models only have differences in geometric shapes to evaluate the performance of the new trapezoidal structure and the previous structures.

**Figure 6:**
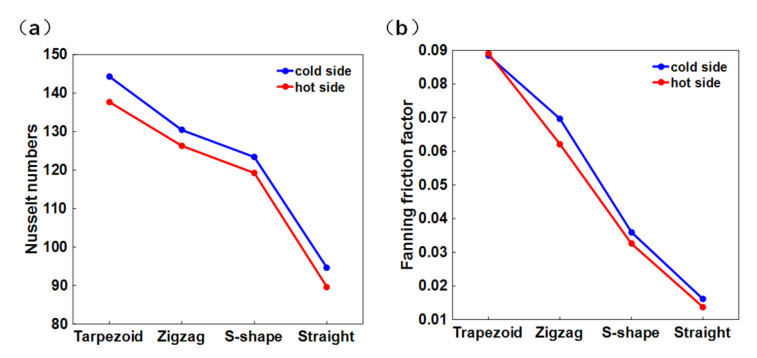
(**a**) The Nusselt numbers and (**b**) the Fanning friction factors of the trapezoidal, the zigzag, the S-shape, and the straight channels.

The trapezoidal structure possesses the largest Nusselt number at the same Reynolds number due to the largest heat exchange area and stronger flow vortex, where its Nusselt number at the hot and the cold sides are 53.62% and 52.47% higher than that of the straight structure, 15.49% and 16.97% higher than that of the S-shape structure, and 9.03% and 10.67% higher than that of the zigzag structure. In all the channel structures, the Nusselt numbers at cold sides are slightly larger than those at hot sides, indicating a low temperature and a high pressure leads to a better heat exchange performance of sCO_2_. However, the trapezoidal channel suffers from the largest flow resistance, as shown in Figure 6b, where its Fanning friction factor is 5 times, 2.5 times, and 1.2 times higher than that of the straight, the S-shape, and the zigzag channels, respectively.

Characteristics of heat transfer and flow resistance are further compared in Table 5 with PEC data obtained by using the trapezoidal structure as the numerator and the previous structures as the denominator. The trapezoidal structure presents a similar overall performance compared with the zigzag structure, and a better property than the S-shaped and the straight structures. This can be attributed to the increase in flow resistance with the enhancement of heat transfer in the trapezoidal structure, indicating a further optimization is still needed.

### 3.3. Optimization of Trapezoidal PCHE

**Figure 7:**
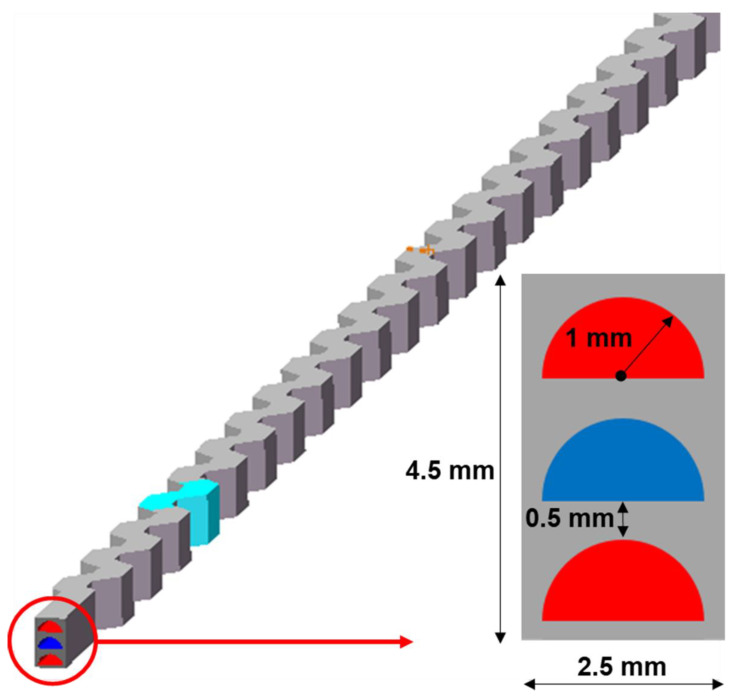
The three-channel model of one cold channel sandwiched between two hot channels.

To reduce the pressure loss and enhance the regenerative efficiency, a sandwiched trapezoidal flow channel structure is designed, as shown in Figure 7, where the cross-section of the flow unit model is increased to 2.5 mm × 4.5 mm with one cold flow channel sandwiched between two hot flow channels. Along with the change in structure, the flow rate in each hot channel is decreased by half, bringing a more sufficient heat exchange.

**Figure 8:**
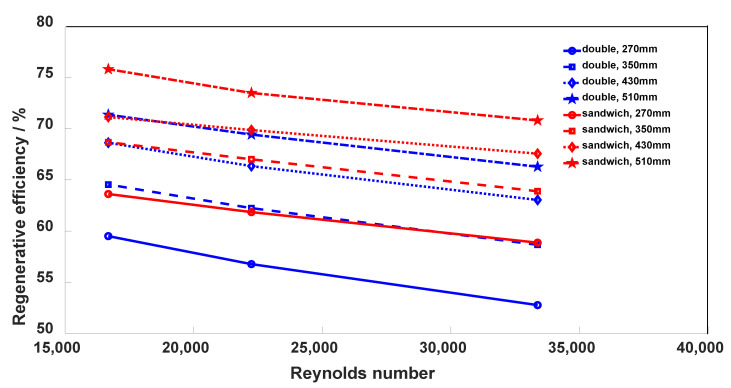
Efficiency comparisons at different Reynolds numbers and flow lengths.

The comparison of pressure loss between the optimized structure and the original design is presented in Table 6. The inlet pressure and temperature conditions are the same with previous simulations, that is 388.75 K, 20.2 MPa in the cold side and 726.85 K, 7.6 MPa in the hot side. The mass flow rates in all cold sides are 14.45 × 10^−3^ kg/s, 9.63 × 10^−4^ kg/s, and 7.23 × 10^−4^ kg/s, corresponding to the Reynolds number of 33,366, 22,244, and 16,683, respectively. As for the hot side flow, it is the same with the cold side in the double-channel structure, and half of the cold side in the sandwich structure because there are two hot channels. Significant reductions in pressure drop loss in the hot channel are obtained, where the values are 75.4% (from 154.81 kPa to 38.37 kPa) and 74.7% (from 39.65 kPa to 10.05 kPa) at 42 kPa and 11 kPa pressure loss cases in the cold side, respectively. The sandwich structure also shows a higher regenerative efficiency, as shown in Figure 8, where the values are increased by 5% at all same length and mass flow conditions.

## 4. Conclusions

In this work, 3D PCHE models were developed with a focus on the trapezoidal flow channels, where its thermal and hydraulic performances with sCO_2_ as the medium were studied. Key factors, including the convective heat transfer coefficient, the regenerative efficiency, and the dimensionless Nusselt numbers, were calculated and analyzed with respect to the changes in geometric parameters, such as the flow channel length, the trapezoidal bottom angle, and the straight length of trapezoidal upper. The Fanning friction factors were further calculated to describe the frictional resistance of different cases. Comparisons of the heat transfer performance and the pressure loss were also discussed between the current structure and previous flow channel types in the literature. We found the Nusselt numbers increased approximately linearly with the increase in Reynolds numbers, but kept almost a constant value at different flow lengths, indicating the independence of heat transfer performance from the flow length. Besides, the thermal and hydraulic performance of the trapezoidal channel changed uniformly with the geometric parameters, where both the Nusselt numbers and Fanning friction factors could be enhanced with the increase in bottom angle and the decrease in upper straight length. Among the four channels, the trapezoidal structure presented the largest increase in Nusselt number in both the hot side and the cold side, and its Fanning friction factor of the trapezoidal channel was 5, 2.5, and 1.2 times higher than that of the straight, S-shaped, and zigzag channels, respectively. By comparing the heat transfer and flow resistances, the overall performance of the trapezoidal structure was found to be close to the zigzag structure, and much better than the S-shaped and the straight structures. Based on the above findings, a sandwiched structure was designed with a couple of hot channels corresponding to one cold channel to optimize the pressure loss in the hot channel and enhance the regenerative efficiency. Through this novel design, the regenerative efficiency could be increased by more than 5% and the pressure drop loss of the hot channel could be reduced by about 75% to only 40 kPa.

## Figures and Tables

**Table 1:** Experimental research on the thermal–hydraulic performance of PCHE.

Structure	Medium	Temperature (℃)	Pressure (MPa)	Flow Rate (kg/h)	Deficiency	Ref.
Straight	Helium	Hot side: 208–790 Cold side: 85–390	Hot side: 1.0–2.7 Cold side:1.0–2.7	15–49	No sCO_2_ Low pressure One channel only	[8]
Straight	Hot side: sCO_2_ Cold side: water	Hot side: 37–102 Cold side: -	Hot side: 8–11 Cold side: -	sCO_2_: 150–650	Not sCO_2_ heat exchange Low temperature One channel only	[10]
Zigzag	CO_2_	Hot side: 280–300 Cold side: 90–108	Hot side: 2.2–3.2 Cold side: 6.5–10.5	40–80	Low pressure under supercritical state One channel only	[2]
Zigzag S-shape	CO_2_	Hot side: 120 Cold side: 35–55	Hot side: 6 Cold side: 7.7–12	40–150	Low pressure at hot side	[3,4]
Zigzag	Hot side: He-CO_2_ Cold side: water	Hot side: 104.5–217.6 Cold side: 23.6–25.2	Hot side: 1.17–1.72 Cold side: 0.104–0.306	He-CO_2_: 154–329 Water: 389–1966	Mixed sCO_2_ Low pressure Low temperature One channel only	[5,6,7]
Zigzag	Hot side: CO_2_ Cold side: water	Hot side: 26–43 Cold side: 15	Hot side: 7.3–8.6 Cold side: -	-	Not sCO_2_ heat exchange Transcritical phase exists Low temperature One channel only	[9]
Airfoil	Hot side: CO_2_ Cold side: water	Hot side: 70–110 Cold side: 16–25	Hot side: 7.6–9.0 Cold side: Approx. 0.1	CO_2_: 500–1800 Water: Approx. 3000	Not sCO_2_ heat exchange Low temperature One channel only	[11]

**Table 2:** Numerical simulation research on the thermal–hydraulic performance of PCHE.

Structure	Medium	Temperature (℃)	Pressure (MPa)	Flow Rate (kg/h)	Deficiency	Ref.
Straight	Hot side: CO_2_ Cold side: water	Hot side: 40–100 Cold side: 14–50	Hot side: 7.5/8.1 Cold side: 0.101325	Hot side: 2.22–8.87 Cold side: 22.17–26.60	Not sCO_2_ heat exchange Low temperature One channel only	[22]
Straight S-shape	sCO_2_	101.85	8	1.44	Not a heat exchanger One side only Low temperature	[23]
Zigzag Airfoil	CO_2_	Hot side: 279.9 Cold side: 107.9	Hot side: 2.52 Cold side: 8.28	Hot side: 0.52 Cold side: 1.13	Low pressure under supercritical state Low temperature	[15]
Zigzag	Helium	Hot side: 800 Cold side: 520	Hot side: 7 Cold side: 7.97	450	No sCO_2_ One channel only	[16]
Zigzag	CO_2_	Hot side: 303.3 Cold side: 61.9	Hot side: 1.9 Cold side: 1.9	-	Low pressure under supercritical state Low temperature One channel only	[17]
Zigzag	CO_2_	Hot side: 280 Cold side: 108	Hot side: 3.2 Cold side: 10.5	30–400	Low pressure under supercritical state Low temperature One channel only	[18]
Zigzag Airfoil	CO_2_	Hot side: 279.9 Cold side: 107.9	Hot side: 2.52 Cold side: 8.28	Hot side: 3.12 Cold side: 3.4	Low pressure under supercritical state Low temperature One channel only	[20]
S-shape	Hot side: LNG flue gas Cold side: sCO_2_	Hot side: 650 Cold side: 224	Hot side: 0.1 Cold side: 13.6	-	Not sCO_2_ heat exchange Low pressure Low temperature One channel only	[19]
S-shape	CO_2_	Hot side: 280 Cold side: 108	Hot side: 2.5 Cold side: 7.4	64.7	Low pressure under supercritical state Low temperature One channel only	[12,13,14]
Zigzag S-shaped Trapezoidal	Helium	Hot side: 900 Cold side: 540	Hot side: 3 Cold side: 3	10–50	No sCO_2_ Low pressure	[21]

**Table 3:** The grid independence verification by grid convergence index (GCI).

Serial Number	Grid Number	The Hot Outlet Temperature (K)	GCI
1	3,780,000	473.37	$GCI_{1,2}=2.42\%$
2	2,780,000	478.32	$GCI_{2,3}=3.66\%$
3	2,080,000	485.94	

**Table 4:** The fitting correlations of both the hot and the cold sides.

Hot Side	
**Physical parameters**	**Correlations**
Density (kg/$m^{3}$)	$\rho =8.5283\times 10^{-9}T^{4}-2.0952\times 10^{-5}T^{3}+0.01942T^{2}-8.175T+1403$
Specific heat (J/(kg K))	$C_{p}=-5.6476\times 10^{-10}T^{5}+1.6674\times 10^{-6}T^{4}-1.9573\times 10^{-3}T^{3}+1.1431T^{2}-332.24T+\text{39,583}$
Thermal conductivity (W/(mK))	$k=7.16\times 10^{-5}T+0.00069$
Viscosity (Pa s)	$\mu =3.65\times 10^{-8}T+6.65\times 10^{-6}$
**Cold side**	
**Physical parameters**	**Correlations**
Density (kg/$m^{3}$)	$\rho =8.878\times 10^{-8}T^{4}-2.0795\times 10^{-4}T^{3}+0.018288T^{2}-72.032T+\text{10,976}$
Specific heat (J/(kg K))	$C_{p}=1.7498\times 10^{-12}T^{6}-7.3206\times 10^{-9}T^{5}+1.2479\times 10^{-5}T^{4}-0.01116T^{3}+5.5477T^{2}-1459.5T+\text{160,650}$
Thermal conductivity (W/(mK))	$k=-4.3538\times 10^{-14}T^{5}+1.2744\times 10^{-10}T^{4}-1.4888\times 10^{-7}T^{3}+8.6829\times 10^{-5}T^{2}-0.025249T+2.9656$
Viscosity (Pa s)	$\mu =2.2325\times 10^{-19}T^{6}-7.5867\times 10^{-16}T^{5}+\text{10,702}\times 10^{-12}T^{4}-8.0234\times 10^{-10}T^{3}+3.3745\times 10^{-7}T^{2}-7.5534\times 10^{-5}T+7.0617\times 10^{-3}$

**Table 5:** The PEC data of four channel structures in both the cold and the hot sides.

PEC-cold	Trapezoid/Zigzag	Trapezoid/S-shape	Trapezoid/Straight
	0.978	1.154	1.157
PEC-hot	Trapezoid/Zigzag	Trapezoid/S-shape	Trapezoid/Straight
	1.034	1.211	1.214

**Table 6:** Pressure loss comparisons between sandwich-structure and double-channel models.

Reynolds Number of the Cold Side	Pressure Loss (kPa)			
	Double-Channel Structure		Sandwich Structure	
	Cold Side	Hot Side	Cold Side	Hot Side
33,366	42.62	154.81	41.94	38.37
22,244	19.73	71.11	18.60	17.77
16,683	11.07	39.65	11.04	10.05
